# Nonlinear relationship between dietary calcium and magnesium intake and peripheral neuropathy in the general population of the United States

**DOI:** 10.3389/fnut.2023.1217465

**Published:** 2023-09-18

**Authors:** Zhe Wu, Xuesong Yang, Zhishen Ruan, Lianlian Li, Jianlin Wu, Bin Wang

**Affiliations:** ^1^The First Clinical College, Shandong University of Traditional Chinese Medicine, Jinan, China; ^2^Department of Vascular Surgery, The First Affiliated Hospital of Shandong University of Traditional Chinese Medicine, Jinan, China; ^3^The Traditional Chinese Medicine College, Shandong University of Traditional Chinese Medicine, Jinan, China

**Keywords:** calcium, magnesium, peripheral neuropathy, restricted cubic spline, nonlinear relationship

## Abstract

**Background:**

Calcium and magnesium are essential minerals that have significant roles in nerve function and regulation. There may be a correlation between dietary calcium and magnesium intake and peripheral neuropathy. However, this relationship remains unclear and requires further study.

**Methods:**

Data from 7,726 participants in the National Health and Nutrition Examination Survey (NHANES) from 1999 to 2004 were analyzed in this study. The relationship between total dietary calcium and magnesium intake, as well as each quantile, and peripheral neuropathy was analyzed using a multifactor logistic regression model. To illustrate the dose–response relationship between calcium and magnesium intake and peripheral neuropathy, we utilized a restricted cubic spline (RCS) plot.

**Results:**

Our analysis found a positive correlation between dietary intake of calcium and magnesium and peripheral neuropathy (calcium: OR 1.000, 95% CI 1.000–1.000; magnesium: OR 1.001, 95% CI 1.00–1.002). Participants in the first and third quantiles of dietary calcium intake had a significantly higher incidence of peripheral neuropathy than those in the second quantile (OR 1.333, 95% CI 1.034–1.719, OR 1.497, 95% CI 1.155–1.941). Those in the first and third quantiles of dietary magnesium intake also had a significantly higher incidence of peripheral neuropathy than those in the second quantile (OR 1.275, 95% CI 1.064–1.528, OR 1.525, 95% CI 1.231–1.890). The restricted cubic spline analysis revealed a U-shaped nonlinear relationship between dietary intake of calcium and magnesium and peripheral neuropathy.

**Conclusion:**

The study found a U-shaped non-linear relationship between dietary calcium and magnesium intake levels and peripheral neuropathy, indicating that both excessive and insufficient intake of calcium and magnesium can increase the incidence of peripheral neuropathy.

## Introduction

Peripheral neuropathy (PN) is marked by various symptoms associated with sensory and motor dysfunction, like numbness, pain, burning sensation, and muscle atrophy (1). It typically starts in the feet and gradually progresses towards the proximal end, rarely affecting the upper limbs alone (2). Peripheral neuropathy is associated with prolonged and stubborn ulcers, higher amputation, and all-cause mortality rates (3). Its incidence is increasing, especially among the elderly, posing a significant public health challenge (4). The disease is linked to various pathological factors, including diabetes, abnormal immune function, exposure to toxins (such as chemotherapy, drugs, and environmental pollution), and abnormal nutritional status (5). Currently, there are no specific drugs or treatments for peripheral neuropathy, and preventing its onset is a focus of ongoing research (6).

Calcium and magnesium are essential micronutrients that play a vital role in regulating nerve function and muscle conduction (7, 8). Studies suggest that an imbalance of calcium and magnesium homeostasis in the body can cause peripheral nerve degeneration and pain (9–11). However, there is limited research on the relationship between dietary calcium and magnesium intake and peripheral neuropathy. Therefore, we aimed to investigate the potential association between dietary calcium and magnesium intake and peripheral neuropathy in the general adult population using NHANES data from 1999 to 2004.

## Methods

### Data source

The NHANES is a nationwide survey organized by the National Center for Health Statistics (NCHS) in the US. It evaluates the health and nutritional status of Americans through a variety of physical exams, laboratory tests, and interviews. The data analyzed in our study was gathered during the 1999–2004 NHANES cycles. The NHANES 1999–2004 research protocol was approved by the NCHS, and no further institutional review committee approval is necessary for this secondary analysis.

**Figure 1:**
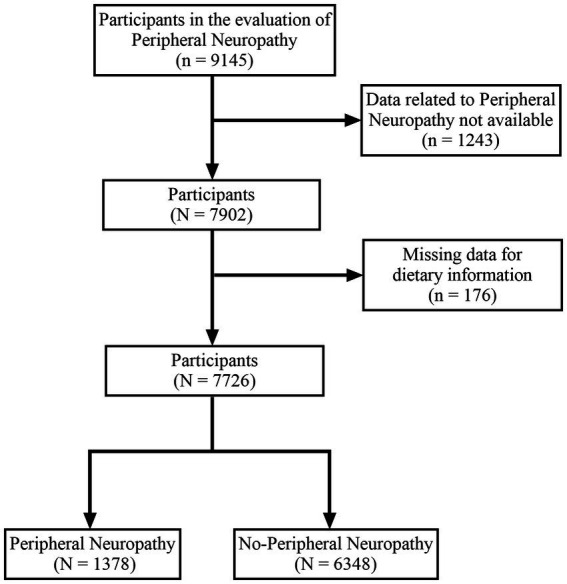
Flow chart of the study.

A total of 9,145 subjects participated in the evaluation of peripheral neuropathy. We excluded subjects who could not provide information on PN (*n* = 1,243) and lacked relevant dietary data (*n* = 176). Ultimately, our study included 7,726 subjects, comprising 1,378 subjects with peripheral neuropathy and 6,348 subjects without peripheral neuropathy (Figure 1).

### Peripheral neuropathy

The health technician used a standard monofilament (Semmes-Weinstein nylon, size 5.07) to apply pressure on three sites of each foot to test the participant’s foot sensation. In case the respondent could not correctly answer or was unsure of the monofilament’s location, the site was considered non-sensory. The presence of peripheral neuropathy was determined as having at least one non-sensory site on both feet (12).

### Dietary intake of calcium and magnesium

The participants’ dietary intake of calcium and magnesium was estimated using the 24 h dietary recall method. This method involves collecting a short and precise list of foods and beverages consumed by the individual over 24 h. The calcium and magnesium content in food is determined by the United States Department of Agriculture’s National Nutrient Database. Trained technicians collect dietary data and conduct nutrient composition analysis to ensure the information’s authenticity and accuracy. The NHANES Dietary Interviewer Procedures Manual provides detailed instructions for this process.

### Covariates

Covariates considered in the analysis were age, gender, race (white, black, Mexican American, or other races), smoking status (now, former, or never), body mass index (BMI), and the presence of cardiovascular disease (CVD), hyperlipidemia, hypertension, diabetes, and chronic kidney disease (CKD). We divided patients with CKD into three groups based on the prognostic risk in the KDIGO 2021 Clinical Practice Guidelines for Glomerular Disease Management: medium risk, high risk, and extremely high risk (13). The diagnostic criteria for the diseases included in the covariates can be found in the attachment (Supplementary material).

### Statistical analysis

We utilized R studio (4.2.1) to analyze NHANES data from 1999 to 2004. All statistical analyses were weighted. Continuous variables were presented as mean ± standard deviation, while categorical variables were expressed as numbers (percentages). We divided the population into three quantiles based on their dietary intake of calcium and magnesium. For calcium intake, the critical values of the three quantiles were: Q1 ≤ 481 mg, 481 mg < Q2 ≤ 843 mg, and Q3 > 843 mg. For magnesium intake, the critical values of the three quantiles were: Q1 ≤ 196 mg, 196 mg < Q2 ≤ 299 mg, and Q3 > 299 mg. The relationship between total dietary calcium and magnesium intake, as well as each quantile, and peripheral neuropathy was analyzed using a multifactor logistic regression model. To illustrate the dose–response relationship between calcium and magnesium intake and peripheral neuropathy, we utilized a restricted cubic spline (RCS) plot.

## Results

### Baseline information

Table 1 presents a comprehensive overview of the characteristics of 7,726 eligible participants, with an average age of 57.15 years, an average dietary calcium intake of 810.83 mg, and an average dietary magnesium intake of 281.31 mg. The participants were categorized into two groups based on the presence or absence of PN. Significant differences were observed between the two groups in terms of age, gender, BMI, smoking status, and the prevalence of hypertension, diabetes, CVD, and prognosis of CKD (*p* < 0.05). However, there was no significant difference between the two groups in terms of race, dietary calcium and magnesium intake, and the prevalence of hyperlipidemia (*p* > 0.05). Analysis of the three quantiles of dietary calcium and magnesium intake (Tables 2, 3) revealed a lower incidence rate of peripheral neuropathy in the second quantile of dietary calcium and magnesium intake compared to the first and third quantiles.

**Table 1:** Baseline characteristics of all subjects.

Variable	Total	No-PN	PN	*p*-value
Age (years)	57.15 ± 0.25	56.03 ± 0.24	64.10 ± 0.52	<0.0001
BMI (kg/m^2^)	28.49 ± 0.12	28.34 ± 0.13	29.47 ± 0.23	<0.0001
Ca (mg)	810.83 ± 10.67	808.27 ± 11.96	826.70 ± 20.99	0.45
Mg (mg)	281.31 ± 3.18	280.07 ± 3.43	289.03 ± 6.83	0.23
Sex				<0.0001
Female	3,882 (50.25%)	3,363 (54.80%)	519 (38.77%)	
Male	3,844 (49.75%)	2,985 (45.20%)	859 (61.23%)	
Race				0.45
White	4,262 (55.16%)	3,506 (78.32%)	756 (76.89%)	
Black	1,384 (17.91%)	1,127 (9.26%)	257 (10.81%)	
Mexican American	1,577 (20.41%)	1,286 (4.40%)	291 (4.55%)	
Other race	503 (6.51%)	429 (8.01%)	74 (7.74%)	
Hyperlipidemia				0.08
No	1,672 (21.64%)	1,381 (21.75%)	291 (18.49%)	
Yes	6,054 (78.36%)	4,967 (78.25%)	1,087 (81.51%)	
CVD				<0.0001
No	6,422 (83.13%)	5,423 (87.58%)	999 (74.09%)	
Yes	1,303 (16.87%)	924 (12.42%)	379 (25.91%)	
Hypertension				<0.0001
No	3,411 (44.17%)	2,938 (51.87%)	473 (39.03%)	
Yes	4,312 (55.83%)	3,408 (48.13%)	904 (60.97%)	
Smoking state				0.02
Former	2,654 (34.4%)	2,109 (33.04%)	545 (39.17%)	
Never	3,592 (46.55%)	2,971 (46.55%)	621 (42.83%)	
Now	1,470 (19.05%)	1,259 (20.41%)	211 (18.00%)	
Diabetes				<0.0001
No	6,294 (81.54%)	5,334 (87.93%)	960 (71.55%)	
Yes	1,425 (18.46%)	1,008 (12.07%)	417 (28.45%)	
Prognosis of CKD				<0.0001
No-CKD	5,533 (75.46%)	4,738 (83.47%)	795 (67.29%)	
Medium risk	1,216 (16.58%)	911 (12.36%)	305 (20.29%)	
High risk	359 (4.90%)	250 (2.82%)	109 (6.77%)	
Extremely high risk	224 (3.06%)	133 (1.35%)	91 (5.65%)	

BMI, body mass index; CVD, cardiovascular disease; CKD, chronic kidney disease.

**Table 2:** Baseline characteristics of all subjects, stratified by the triple quantile of calcium intake.

Variable	Total	Q1	Q2	Q3	*p*-value
Age (years)	57.15 ± 0.25	57.90 ± 0.34	58.06 ± 0.36	55.77 ± 0.32	<0.0001
BMI (kg/m^2^)	28.49 ± 0.12	28.63 ± 0.19	28.54 ± 0.19	28.35 ± 0.20	0.55
Sex					<0.0001
Female	3,882 (50.25%)	1,507 (62.79%)	1,342 (53.57%)	1,033 (43.63%)	
Male	3,844 (49.75%)	1,072 (37.21%)	1,230 (46.43%)	1,542 (56.37%)	
Race					<0.0001
White	4,262 (55.16%)	1,134 (68.40%)	1,441 (78.13%)	1,687 (85.83%)	
Black	1,384 (17.91%)	697 (15.53%)	423 (9.31%)	264 (4.82%)	
Mexican American	1,577 (20.41%)	542 (4.84%)	534 (4.70%)	501 (3.86%)	
Other race	503 (6.51%)	206 (11.22%)	174 (7.87%)	123 (5.50%)	
Hyperlipidemia					0.19
No	1,672 (21.64%)	534 (20.08%)	526 (20.85%)	612 (22.64%)	
Yes	6,054 (78.36%)	2045 (79.92%)	2046 (79.15%)	1963 (77.36%)	
CVD					0.004
No	6,422 (83.13%)	2094 (83.64%)	2,136 (85.41%)	2,192 (87.59%)	
Yes	1,303 (16.87%)	485 (16.36%)	436 (14.59%)	382 (12.41%)	
Hypertension					0.002
No	3,411 (44.17%)	1,013 (46.22%)	1,131 (49.36%)	1,267 (53.79%)	
Yes	4,312 (55.83%)	1,566 (53.78%)	1,440 (50.64%)	1,306 (46.21%)	
Smoking state					<0.0001
Former	2,654 (34.4%)	805 (28.58%)	894 (34.82%)	955 (37.31%)	
Never	3,592 (46.55%)	1,202 (47.19%)	1,236 (46.61%)	1,154 (44.62%)	
Now	1,470 (19.05%)	570 (24.23%)	437 (18.58%)	463 (18.07%)	
Diabetes					0.02
No	6,294 (81.54%)	2061 (84.65%)	2090 (84.22%)	2,143 (87.68%)	
Yes	1,425 (18.46%)	515 (15.35%)	480 (15.78%)	430 (12.32%)	
PN					0.15
No	6,348 (82.16)	2,105 (85.39)	2,138 (87.79)	2,105 (85.25)	
Yes	1,378 (17.84)	474 (14.61)	434 (12.21)	470 (14.75)	
CKD					<0.0001
No-CKD	5,533 (74.55%)	1747 (75.74%)	1834 (80.01%)	1952 (84.53%)	
Medium risk	1,216 (16.58%)	435 (16.03%)	428 (13.83%)	353 (11.13%)	
High risk	359 (4.9%)	149 (4.81%)	119 (3.36%)	91 (2.25%)	
Extremely high risk	224 (3.06%)	93 (2.58%)	74 (2.11%)	57 (1.31%)	

BMI, body mass index; CVD, cardiovascular disease; CKD, chronic kidney disease; PN, peripheral neuropathy.

**Table 3:** Baseline characteristics of all subjects, stratified by the triple quantile of magnesium intake.

Variable	Total	Q1	Q2	Q3	*p*-value
Age (years)	57.15 ± 0.25	58.75 ± 0.38	57.56 ± 0.34	55.51 ± 0.28	<0.0001
BMI (kg/m^2^)	28.49 ± 0.12	28.72 ± 0.18	28.73 ± 0.18	28.11 ± 0.19	0.01
Sex					<0.0001
Female	3,882 (50.25%)	1,682 (69.45%)	1,345 (56.03%)	855 (36.11%)	
Male	3,844 (49.75%)	905 (30.55%)	1,224 (43.97%)	1,715 (63.89%)	
Race					<0.0001
White	4,262 (55.16%)	1,199 (69.88%)	1,474 (79.79%)	1,589 (83.24%)	
Black	1,384 (17.91%)	678 (15.15%)	401 (8.55%)	305 (5.76%)	
Mexican American	1,577 (20.41%)	544 (4.98%)	516 (4.04%)	517 (4.32%)	
Other race	503 (6.51%)	166 (9.99%)	178 (7.62%)	159 (6.68%)	
Hyperlipidemia					0.37
No	1,672 (21.64%)	523 (20.04%)	547 (21.75%)	602 (21.90%)	
Yes	6,054 (78.36%)	2,064 (79.96%)	2,022 (78.25%)	1,968 (78.10%)	
CVD					0.01
No	6,422 (83.13%)	2,081 (83.59%)	2,149 (85.44%)	2,192 (87.62%)	
Yes	1,303 (16.87%)	506 (16.41%)	420 (14.56%)	377 (12.38%)	
Hypertension					<0.0001
No	3,411 (44.17%)	974 (43.83%)	1,126 (49.30%)	1,311 (55.77%)	
Yes	4,312 (55.83%)	1,612 (56.17%)	1,441 (50.70%)	1,259 (44.23%)	
Smoking state					<0.0001
Former	2,654 (34.4%)	779 (27.85%)	886 (33.97%)	989 (38.65%)	
Never	3,592 (46.55%)	1,248 (47.31%)	1,211 (47.10%)	1,133 (44.08%)	
Now	1,470 (19.05%)	555 (24.84%)	471 (18.94%)	444 (17.28%)	
Diabetes					0.16
No	6,294 (81.54%)	2,066 (84.70%)	2,091 (85.15%)	2,137 (86.86%)	
Yes	1,425 (18.46%)	518 (15.30%)	476 (14.85%)	431 (13.14%)	
PN					0.002
No	6,348 (82.16%)	2,094 (85.57%)	2,146 (88.41%)	2,108 (84.54%)	
Yes	1,378 (17.84%)	493 (14.43%)	423 (11.59%)	462 (15.46%)	
CKD					<0.0001
No-CKD	5,533 (74.55%)	1,708 (74.01%)	1,842 (80.88%)	1,983 (85.15%)	
Medium risk	1,216 (16.58%)	453 (16.45%)	418 (13.47%)	345 (11.12%)	
High risk	359 (4.9%)	160 (5.35%)	127 (3.39%)	72 (1.80%)	
Extremely high risk	224 (3.06%)	106 (3.12%)	67 (1.61%)	51 (1.33%)	

BMI, body mass index; CVD, cardiovascular disease; CKD, chronic kidney disease; PN, peripheral neuropathy.

### The relationship between dietary calcium and magnesium intake and peripheral neuropathy

After adjusting for all covariates, the multifactor logistic regression analysis (Table 4) revealed a positive correlation between dietary calcium and magnesium intake and peripheral neuropathy (calcium: OR: 1.000, 95%CI: 1.000–1.000; magnesium: OR: 1.001, 95%CI: 1.000–1.002). When dietary calcium and magnesium intake were divided into three quantiles, it was observed that the incidence rate of peripheral neuropathy was significantly higher in the first and third quantiles of dietary calcium intake compared to the second quantile (OR 1.333, 95% CI 1.034–1.719, OR 1.497, 95% CI 1.155–1.941). The incidence of peripheral neuropathy in the first and third quantiles of dietary magnesium intake was also significantly higher than that in the second quantiles (OR 1.275, 95% CI 1.064–1.528, OR 1.525, 95% CI 1.231–1.890). This suggests the possibility of a non-linear relationship between dietary calcium and magnesium intake and peripheral neuropathy.

**Table 4:** Relationship between dietary calcium and magnesium intake and peripheral neuropathy.

Result	Model 1		Model 2		Model 3	
	OR (95%CI)	*p*-value	OR (95%CI)	*p*-value	OR (95%CI)	*p*-value
Calcium	1.000 (1.000,1.000)	0.444	1.000 (1.000,1.000)	0.160	1.000 (1.000,1.000)	0.050
Q2	ref	ref	ref	ref	ref	ref
Q1	1.230 (0.987,1.532)	0.065	1.311 (1.037,1.659)	0.025	1.333 (1.034,1.719)	0.028
Q3	1.244 (0.995,1.555)	0.056	1.377 (1.074,1.765)	0.013	1.497 (1.155,1.941)	0.004
Magnesium	1.001 (1.000,1.001)	0.214	1.001 (1.000,1.001)	0.172	1.001 (1.000,1.002)	0.037
Q2	ref	ref	ref	ref	ref	ref
Q1	1.287 (1.083,1.531)	0.005	1.322 (1.107,1.579)	0.003	1.275 (1.064,1.528)	0.010
Q3	1.395 (1.145,1.699)	0.001	1.438 (1.175,1.760)	<0.001	1.525 (1.231,1.890)	<0.001

Adjusted variables: Model 1: unadjusted. Model 2: age, sex, and race. Model 3: all. OR, odds ratio; CI, confidence interval.

### RCS

**Figure 2:**
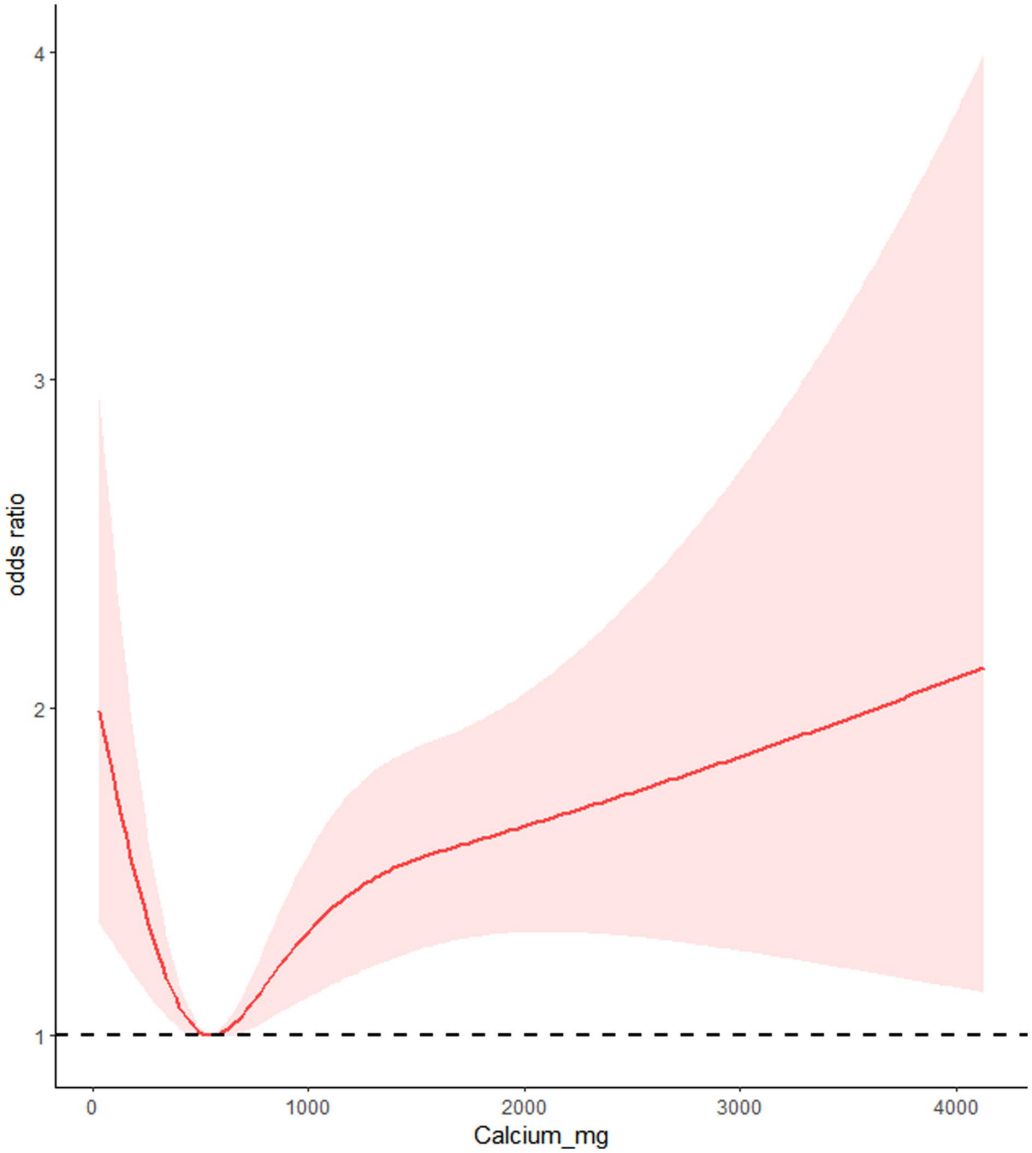
Restrictive cubic spline of dietary calcium intake and peripheral neuropathy.

**Figure 3:**
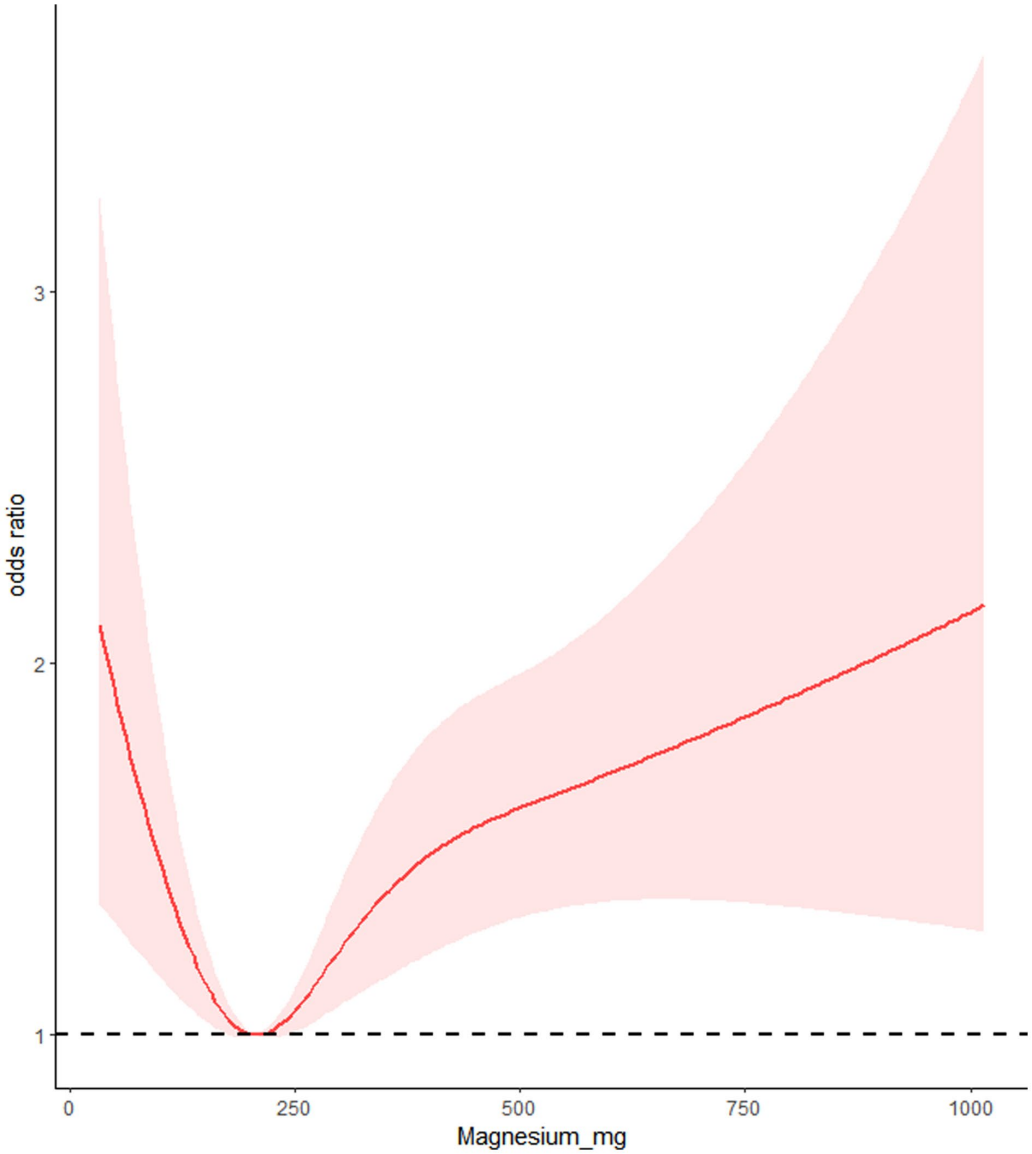
Restrictive cubic spline of dietary magnesium intake and peripheral neuropathy.

We utilized RCS to analyze the relationship between dietary calcium and magnesium intake and peripheral neuropathy. The RCS results revealed a U-shaped nonlinear relationship between dietary calcium and magnesium and peripheral neuropathy (*p* for nonlinear <0.001). The critical point of the U-shaped curve for dietary calcium intake and peripheral neuropathy was determined to be 549.72 mg. When dietary calcium intake was below 549.72 mg, there was a negative correlation between dietary calcium intake and the incidence of peripheral neuropathy. However, when dietary calcium intake exceeded 549.72 mg, there was a positive correlation between dietary calcium intake and the incidence of peripheral neuropathy. The critical point of the U-shaped curve for dietary magnesium intake and peripheral neuropathy was determined to be 206.53 mg. When magnesium intake was below 206.53 mg, there was a negative correlation between dietary magnesium intake and the incidence of peripheral neuropathy. When magnesium intake exceeded 206.53 mg, there was a positive correlation between dietary magnesium intake and the incidence of peripheral neuropathy (Figures 2, 3).

### Threshold effect analysis

After adjusting for all covariates, the results of the stratification analysis (Table 5) showed an overall negative correlation trend between dietary calcium and magnesium intake and peripheral neuropathy when dietary calcium and magnesium intake did not exceed the critical values (calcium: OR: 0.999, 95%CI: 0.998–1.000; magnesium: OR: 0.996, 95%CI: 0.991–1.001). When dietary calcium and magnesium intake exceeded the critical value, there was a significant positive correlation between dietary calcium and magnesium intake and peripheral neuropathy (calcium: OR: 1.000, 95%CI: 1.000–1.000; magnesium: OR: 1.001, 95%CI: 1.000–1.002).

**Table 5:** Threshold effect analysis.

Character	OR (95% CI)	*p*-value
Calcium		
≧549.72 mg	1.000 (1.000,1.000)	<0.001
<549.72 mg	0.999 (0.998,1.000)	0.116
Magnesium		
≧206.53 mg	1.001 (1.000,1.002)	0.019
<206.53 mg	0.996 (0.991,1.001)	0.114

OR, odds ratio; CI, confidence interval.

## Discussion

In this cross-sectional study, we aimed to investigate the relationship between dietary intake levels of calcium and magnesium and peripheral neuropathy in the general population of the United States. Our findings showed a U-shaped nonlinear relationship between dietary intake levels of calcium and magnesium and peripheral neuropathy, indicating that both excessive and insufficient intake of these minerals can increase the incidence of peripheral neuropathy. To our knowledge, this is the first study to explore the association between dietary intake levels of calcium and magnesium and peripheral neuropathy in the general population.

Previous research on the relationship between calcium and magnesium and peripheral neuropathy has not been consistent, especially in cancer patients (14–16). While some studies have reported that calcium and magnesium infusion can reduce the incidence and severity of peripheral neuropathy after chemotherapy (15, 17), more studies have reported negative results (18–20). In addition, Wesselink et al. found that magnesium intake is associated with the incidence of chemotherapy-induced peripheral neuropathy in cancer patients, while calcium intake is not (21). But, research on the relationship between calcium and magnesium and peripheral neuropathy in non-cancer populations is limited, with no reports on the relationship between dietary intake of calcium and magnesium and peripheral neuropathy in the general population.

Calcium and magnesium are both essential minerals that play important roles in the function and regulation of peripheral nerves (22–24). Calcium and magnesium play a critical role in maintaining the stability and excitability of the nerve cell (25, 26). Calcium and magnesium homeostasis helps maintain appropriate membrane potential, thus ensuring the transmission of nerve impulses. They also regulate signal transduction within nerve cells, with calcium ions controlling the permeability of potassium and sodium ions, and magnesium ions potentially affecting calcium ion signal transduction (27). In addition, calcium and magnesium participate in various neural cell metabolic processes, including protein synthesis and decomposition, enzyme activation, and energy metabolism, which impact the survival and function of neural cells (28, 29). Proper levels of calcium and magnesium also help maintain the pH value within nerve cells. Therefore, deficiency or excess of calcium or magnesium can affect the function and regulation of peripheral nerves.

Peripheral neuropathy is commonly associated with diabetes (30), but our research suggests that diabetes accounts for only 28.45% of all cases of peripheral neuropathy. This suggests that neuropathy caused by other factors may be significantly underdiagnosed or overlooked (31). In addition, it is important to note that the co-occurrence of peripheral neuropathy with other chronic diseases such as hypertension, CVD, and CKD could potentially complicate the management and treatment of peripheral neuropathy. In this study, 60.97% of the subjects with peripheral neuropathy also suffered from hypertension, 25.91% of the patients also suffered from CVD. It also indicates that peripheral neuropathy may be related to other chronic diseases except diabetes.

We must acknowledge the study’s limitations. One of the primary constraints is that this is a cross-sectional study, and as such, it cannot establish the underlying reasons for the observed non-linear relationship between dietary calcium and magnesium intake and peripheral neuropathy. Additionally, the use of the 24 h recall method to determine dietary calcium and magnesium intake may not accurately reflect long-term calcium and magnesium intake.

## Conclusion

Our study revealed a U-shaped-nonlinear correlation between dietary calcium and magnesium intake levels and peripheral neuropathy in the general population of the United States. These findings suggest that maintaining appropriate levels of calcium and magnesium intake could potentially aid in preventing peripheral neuropathy. Nevertheless, further research is required to validate these outcomes and examine the potential mechanisms underlying the association between dietary calcium and magnesium intake and peripheral neuropathy.

## Data availability statement

The raw data supporting the conclusions of this article will be made available by the authors, without undue reservation.

## Ethics statement

NHANES is a nationwide survey organized by the National Center for Health Statistics (NCHS) in the US. The NHANES 1999–2004 research protocol was approved by the NCHS, and no further institutional review committee approval is necessary for this secondary analysis. The patients/participants provided their written informed consent to participate in this study.

## Author contributions

ZW led the study design and data collection. XY, ZR, and LL contributed to the interpretation of the results. BW and JW contributed to the manuscript writing. All authors contributed to the article and approved the submitted version.
