# Supplementary material for: Nonlinear relationship between dietary calcium and magnesium intake and peripheral neuropathy in the general population of the United States
Source: Front Nutr. 2023 Sep 18;10:1217465. doi: 10.3389/fnut.2023.1217465 (PMC10544963; doi:10.3389/fnut.2023.1217465)
Supplement: Supplementary file 1 [file Data_Sheet_1.docx]

Diagnostic criteria for diseases

1. Hypertension is defined as an average systolic blood pressure of ≥ 140 millimeters of mercury and an average diastolic blood pressure of ≥ 90 millimeters of mercury, diagnosed by a doctor, or using antihypertensive drugs.
2. Diabetes is defined as fasting blood glucose ≥ 7 mmol/L or random blood glucose ≥ 11.1 mmol/L or two-hour OGTT blood glucose ≥ 11.1 mmol/L or glycosylated hemoglobin>6.5%, or diagnosed by a doctor, or using hypoglycemic drugs.
3. Hyperlipidemia is defined as triglyceride ≥ 150mg/dL, total cholesterol ≥ 200mg/dL or low-density lipoprotein ≥ 130mg/dL, male high-density lipoprotein ≤ 40mg/dL or female high-density lipoprotein ≤ 50mg/dL, or the use of lipid-lowering drugs.
4. Cardiovascular disease (CVD) outcomes are defined as a composite of five self reported CVD outcomes, including congestive heart failure, coronary heart disease, angina, heart attack, and stroke. If the participant answers' yes' to the following question, it is recorded as having CVD: “Have doctors or other health professionals ever told you that you have congestive heart failure/coronary heart disease/angina pectoris/stroke?”
5. Chronic kidney disease (CKD) was defined as estimated glomerular filtration rate (eGFR) < 60 ml/min/1.73m^2^ or as urinary albumin-to-creatinine ratio (ACR) ≥ 30 mg/g.

Medium risk was defined as estimated glomerular filtration rate 45 ≤ (eGFR) < 60 ml/min/1.73m^2^ and urinary albumin-to-creatinine ratio (ACR) ≥ 30 mg/g, or estimated glomerular filtration rate 60 ≤ (eGFR) < 90 ml/min/1.73m^2^ and urinary albumin-to-creatinine ratio (ACR) < 30 mg/g.

High risk was defined as estimated glomerular filtration rate 30 ≤ (eGFR) < 45 ml/min/1.73m^2^ and urinary albumin-to-creatinine ratio (ACR) < 30 mg/g, or estimated glomerular filtration rate 45 ≤ (eGFR) < 60 ml/min/1.73m^2^ and 30 ≤ urinary albumin-to-creatinine ratio (ACR) ≤ 300 mg/g, or estimated glomerular filtration rate 60 ≤ (eGFR) < 90 ml/min/1.73m^2^ and urinary albumin-to-creatinine ratio (ACR) > 300 mg/g,

Extremely high risk was defined as estimated glomerular filtration rate 45 ≤ (eGFR) < 60 ml/min/1.73m^2^ and urinary albumin-to-creatinine ratio (ACR) > 300 mg/g, or estimated glomerular filtration rate 30 ≤ (eGFR) < 45 ml/min/1.73m^2^ and urinary albumin-to-creatinine ratio (ACR) ≥ 30 mg/g, or estimated glomerular filtration rate (eGFR) < 30 ml/min/1.73m^2^.
